# Race and sex differences in dropout from the STRRIDE trials

**DOI:** 10.3389/fspor.2023.1215704

**Published:** 2023-07-10

**Authors:** Katherine A. Collins, Kim M. Huffman, Ruth Q. Wolever, Patrick J. Smith, Ilene C. Siegler, Leanna M. Ross, John M. Jakicic, Paul T. Costa, William E. Kraus

**Affiliations:** ^1^ Duke Molecular Physiology Institute, Duke University School of Medicine, Durham, NC, United States; ^2^ Division of Rheumatology, Duke University School of Medicine, Durham, NC, United States; ^3^ Department of Physical Medicine and Rehabilitation, Vanderbilt University School of Medicine, Nashville, TN, United States; ^4^ Department of Psychiatry, University of North Carolina at Chapel Hill School of Medicine, Chapel Hill, NC, United States; ^5^ Department of Psychiatry and Behavioral Sciences, Duke University School of Medicine, Durham, NC, United States; ^6^ Division of Cardiology, Duke University School of Medicine, Durham, NC, United States; ^7^ Department of Internal Medicine, University of Kansas Medical Center, Kansas City, KS, United States; ^8^ Department of Medicine, Division of Geriatrics, Duke University School of Medicine, Durham, NC, United States

**Keywords:** non-compliance, lifestyle intervention, adherence, health disparity, retention

## Abstract

**Purpose:**

To determine if race and sex differences exist in determinants and timing of dropout among individuals enrolled in an exercise and/or caloric restriction intervention.

**Methods:**

A total of 947 adults with dyslipidemia (STRRIDE I, STRRIDE AT/RT) or prediabetes (STRRIDE-PD) were randomized to either inactive control or to 1 of 10 exercise interventions, ranging from doses of 8–23 kcal/kg/week, intensities of 50%–75% $\dot{V}O_{2}$ peak, and durations of 6–8 months. Two groups included resistance training, and one included a dietary intervention (7% weight loss goal). Dropout was defined as an individual withdrawn from the study, with the reasons for dropout aggregated into determinant categories. Timing of dropout was defined as the last session attended and aggregated into phases (i.e., “ramp” period to allow gradual adaptation to exercise prescription). Utilizing descriptive statistics, percentages were generated according to categories of determinants and timing of dropout to describe the proportion of individuals who fell within each category.

**Results:**

Black men and women were more likely to be lost to follow-up (Black men: 31.3% and Black women: 19.6%), or dropout due to work responsibilities (15.6% and 12.5%), “change of mind” (12.5% and 8.9%), transportation issues (6.3% and 3.6%), or reported lack of motivation (6.3% and 3.6%). Women in general noted lack of time more often than men as a reason for dropout (White women: 22.4% and Black women: 22.1%). Regardless of race and sex, most participants dropped out during the ramp period of the exercise intervention; with Black women (50%) and White men (37.1%) having the highest dropout rate during this period.

**Conclusion:**

These findings emphasize the importance of targeted retention strategies when aiming to address race and sex differences that exist in determinants and timing of dropout among individuals enrolled in an exercise and/or caloric restriction intervention.

## Introduction

Most adults recognize the positive health benefits of being physically active and maintaining a healthy weight, yet physical inactivity and obesity rates continue to rise (1, 2). This dissonance is even more pronounced among individuals of differing race and sex across the United States. While in non-Hispanic White adults, the prevalence rates of obesity and physical inactivity are 41.4% and 23.0%, non-Hispanic Black adults exhibit greater rates at 49.9% and 30.0%, respectively (1). Moreover, as compared to all other race and sex classifications, the greatest prevalence of both obesity and physical inactivity is found in Black women (1).

Numerous exercise studies—alone and in combination with caloric restriction—are efficacious for improving body weight, body composition, and fitness, as well as preventing or reducing the risk for chronic disease (1–6). While these effective strategies exist, many individuals either fail to complete these interventions (i.e., dropout) or fail to adhere long-term to these recommended behavior changes (7–12). Therefore, understanding factors that influence exercise intervention dropout and the timing of dropout is of importance. Moreover, identifying race and sex differences in determinants and timing of dropout can inform the development of more precise approaches for optimizing exercise adoption and adherence. This in turn, can improve the risk for chronic disease development in the United States as a whole.

The three STRRIDE (Studies of a Targeted Risk Reduction Intervention through Defined Exercise) randomized trials investigated the effects of exercise amount, mode, intensity, and weight loss (via caloric restriction) on cardiometabolic health among adults with overweight or obesity. Recently we reported on differences in baseline characteristics amongst individuals who dropped out vs. completed the STRRIDE trials—one of the key differences noted was the proportion of non-White participants, as well as a nearly significant difference in the proportion of women participants (7). These findings laid the foundation to further explore the race and sex differences in determinants and timing of dropout that may exist among individuals initially motivated to participate in a supervised exercise and/or caloric restriction intervention.

## Materials and methods

### Study participants

Race and sex differences in determinants and timing of dropout were assessed in participants from STRRIDE I (10), STRRIDE AT/RT (11), and STRRIDE-PD (12). STRRIDE I (1999–2003) and STRRIDE AT/RT (2004–2008) enrolled previously sedentary adults with overweight or obesity and mild-to-moderate dyslipidemia (classified by LDL-cholesterol 130–190 mg/dl or HDL-cholesterol ≤40 mg/dl for men and ≤45 mg/dl for women). Participants were enrolled at either Duke University Medical Center or East Carolina University (ECU). STRRIDE-PD (2009–2012) enrolled previously sedentary adults with overweight or obesity and prediabetes (defined as two consecutive fasting glucose concentrations ≥95 to <126 mg/dl taken 1 week apart). Participants in STRRIDE-PD were enrolled only at Duke University Medical Center.

Table 1 describes the randomized exercise intervention groups across the three STRRIDE trials (10–12). Both STRRIDE I and AT/RT study protocols were approved by the institutional review boards at Duke University and ECU. The STRRIDE-PD study protocol was approved by the institutional review board at Duke University only. All participants provided both verbal and signed, written informed consent. Baseline demographic characteristics were collected upon enrollment into one of the STRRIDE trials.

**Table 1:** STRRIDE randomized exercise intervention groups.

Intervention group	Exercise prescription	
STRRIDE I		
Inactive Control	–	–
High-Amount/Vigorous-Intensity	23 KKW or 20 miles/week	65%–80% peak $\dot{V}O_{2}$
Low-Amount/Vigorous-Intensity	14 KKW or 12 miles/week	65%–80% peak $\dot{V}O_{2}$
Low-Amount/Moderate-Intensity	14 KKW or 12 miles/week	40%–55% peak $\dot{V}O_{2}$
STRRIDE II		
Aerobic Training (Low-Amount/Vigorous-Intensity)	14 KKW or 12 miles/week	65%–80% peak $\dot{V}O_{2}$
Resistance Training	3 days/week, 3 sets/day, 8–12 reps of 8 exercises	
Aerobic + Resistance Training	14 KKW or 12 miles/week at 65%–80% peak $\dot{V}O_{2}$ + 3 days/week, 3 sets/day, 8–12 reps of 8 exercises	
STRRIDE III		
High-Amount/Vigorous-Intensity	16 KKW or 13.8 miles/week	65%–80% peak $\dot{V}O_{2}$
High-Amount/Moderate-Intensity	16 KKW or 13.8 miles/week	40%–55% peak $\dot{V}O_{2}$
Low-Amount/Moderate-Intensity	10 KKW or 8.6 miles/week	40%–55% peak $\dot{V}O_{2}$
Combined Lifestyle Intervention	10 KKW or 8.6 miles/week at 40%–55% peak $\dot{V}O_{2}$_E_ **+** DIET to reduce 7% body weight	

KKW, kcal/kilogram of body weight/week.

### Intervention details

Study design differences existed across the three STRRIDE trials. In STRRIDE I, no inactive control period “run-in” was conducted. Once randomized, to allow for gradual adaptation to the exercise prescription, participants underwent a ramp period of two to three months. The ramp period was followed by six additional months of training at the appropriate exercise prescription. Prescribed exercise intensity was based on each participant's baseline cardiopulmonary exercise test results. Aerobic exercise modes included treadmills, elliptical trainers, cycle ergometers, or any combination of these.

In STRRIDE AT/RT, participants completed a four-month inactive control period (run-in) before exercise intervention randomization. After randomization, participants underwent an eight- to ten-week ramp period, to allow for gradual adaptation to their exercise prescription. The ramp period was followed by five to six additional months of training at the appropriate exercise prescription. For the aerobic training groups, prescribed exercise intensity was based on each participant's baseline cardiopulmonary exercise test. Aerobic exercise modes included treadmill, elliptical trainer, cycle ergometer, or any combination of these. For the resistance training groups, participants' ramped period began with one set during weeks 1–2, two sets during weeks 3–4, and built up to the three-set prescription on week 5. Resistance exercises included upper body [(bench press, military (or overhead) press, latissimus dorsi pulldown, seated row, and back extension (or biceps flexion and triceps extension)], and lower body (leg extension, leg flexion, and leg press).

In STRRIDE-PD, participants completed a three-month inactive control period (run-in) before exercise intervention randomization. After randomization, participants underwent approximately a ten-week ramp period, to allow gradual adaptation to their exercise prescription; however, the total duration of the exercise intervention was six months, regardless of the duration of the ramp period. Prescribed exercise intensity was based on each participant's baseline cardiopulmonary exercise test. Aerobic exercise modes included treadmill, elliptical trainer, cycle ergometer, or any combination of these. The combined lifestyle group in STRRIDE-PD received an intervention modeled after the Diabetes Prevention Program (13). This group was designed to achieve 7% weight loss via energy intake restriction, a low-fat diet, and exercise. Participants attended four initial group counseling sessions, followed by twelve biweekly intensive behavioral group sessions adapted from the Diabetes Prevention Program.

Across all three STRRIDE trials, intensity and duration for aerobic exercise sessions were verified by direct supervision and/or with the use of downloadable heart rate monitoring (Polar Electro, Woodbury, NY). Resistance training sessions were verified by direct supervision and/or the FitLinxx Strength Training Partner (FitLinxx, Norwalk, CT). The “training partner” automatically sent data from each session to the FitLinxx server computer.

### Dropout

Dropout across the three STRRIDE studies was defined as an individual who was withdrawn from the study because of personal factors, was withdrawn from the study by the principal investigator (PI; e.g., participant intentionally losing weight in STRRIDE I), or lost to follow-up. All Duke and ECU participants who dropped out were included in the denominators for each determinant category. The following categories were created to define determinants for participant dropout:

1) lack of time, 2) transportation issue, 3) biopsy issue (*vastus lateralis* needle biopsies were performed at baseline and intervention conclusion), 4) changed mind, 5) health issue, 6) exacerbation of prior injury, 7) moved/relocated, 8) withdrawn by PI, 9) lost to follow-up, 10) family/caregiving responsibilities, 11) reported lack of motivation, 12) work responsibilities, and 13) travel.

Timing of intervention dropout was defined as the last attended session, whether an assessment or exercise session. The number of individuals were aggregated within each dropout category. Because data from ECU were not entered into an electronic database, we were unable to properly identify timing of intervention dropout among the ECU participants; thus, only individuals participating at the Duke site were included in analyses involving timing of exercise intervention dropout. Based on the last attended session, the timing of dropout was categorized as follows for description purposes:

*Before exercise initiation:* a) baseline visits, b) run-in period;

*During exercise participation:* c) ramp period, d) month 1 of the exercise intervention,

e) month 2, f) month 3, g) month 4, h) month 5, i) month 6, j) month 7; and

*After exercise participation:* k) post-intervention.

### Statistical analyses

This secondary descriptive analysis was conducted using JMP Pro v16.1 (SAS Institute, Cary, NC). Baseline characteristic differences between race (Black vs. White) and sex (men vs. women) were assessed using a two-tailed *t*-test for independent groups. A *p-*value of <0.05 was considered significant. Given lack of power and multiple comparisons, the remaining analyses were descriptive comparisons only. To describe the proportion of individuals who fell within each category, percentages were generated according to dropout determinants and timing.

## Results

Of the 947 participants enrolled 295 (31%) dropped out of the STRRIDE trials. Among participants who dropped out 287 (97%) classified their race as either Black (*n* = 88) or White (*n* = 199); of these participants 115 were men and 172 were women. Table 2 displays baseline characteristics by race and sex. Compared to their White-counterparts, Black participants at baseline were on average younger (48.9 ± 8.6 vs. 54.9 ± 9.1 years of age; *p* < 0.001) and had greater body mass indexes (31.8 ± 3.3 vs. 30.8 ± 3.3 kg/m^2^; *p* = 0.02); however, they had similar cardiorespiratory fitness (24.1 ± 6.0 vs. 25.3 ± 6.0 ml/kg/min; *p* = 0.13). Men had greater cardiorespiratory fitness compared to women (29.2 ± 5.9 vs. 21.8 ± 3.9 ml/kg/min; *p* < 0.001), however they were similar in age (52.4 ± 10.4 vs. 53.5 ± 8.6 years of age; *p* = 0.31) and body mass index (31.0 ± 3.1 vs. 31.2 ± 3.4 kg/m^2^; *p* = 0.65), respectively.

**Table 2:** Baseline characteristics by race and sex among participants who dropped out from the STRRIDE trials.

Baseline characteristics	Race			Sex		
	Black	White	*p*-value	Men	Women	*p*-value
*n = 287*	88 (30.7%)	199 (69.3%)	–	115 (40.1%)	172 (59.9%)	–
Female, *n* (%)	56 (63.6%)	116 (58.3%)	–	–	–	–
White, *n* (%)	–	–	–	83 (72.3%)	116 (67.4%)	–
Age, years	48.9 (8.6)	54.9 (9.1)	<0.0001	52.4 (10.4)	53.5 (8.6)	0.313
Body Mass Index, kg/m^2^	31.8 (3.3)	30.8 (3.3)	0.023	31.0 (3.1)	31.2 (3.4)	0.653
Relative VO_2_, ml/kg/min	24.1 (6.0)	25.3 (6.0)	0.125	29.2 (5.9)	21.8 (3.9)	<0.0001

Values are mean (standard deviation) unless otherwise reported.

### Race differences in dropout determinants and timing

**Figure 1:**
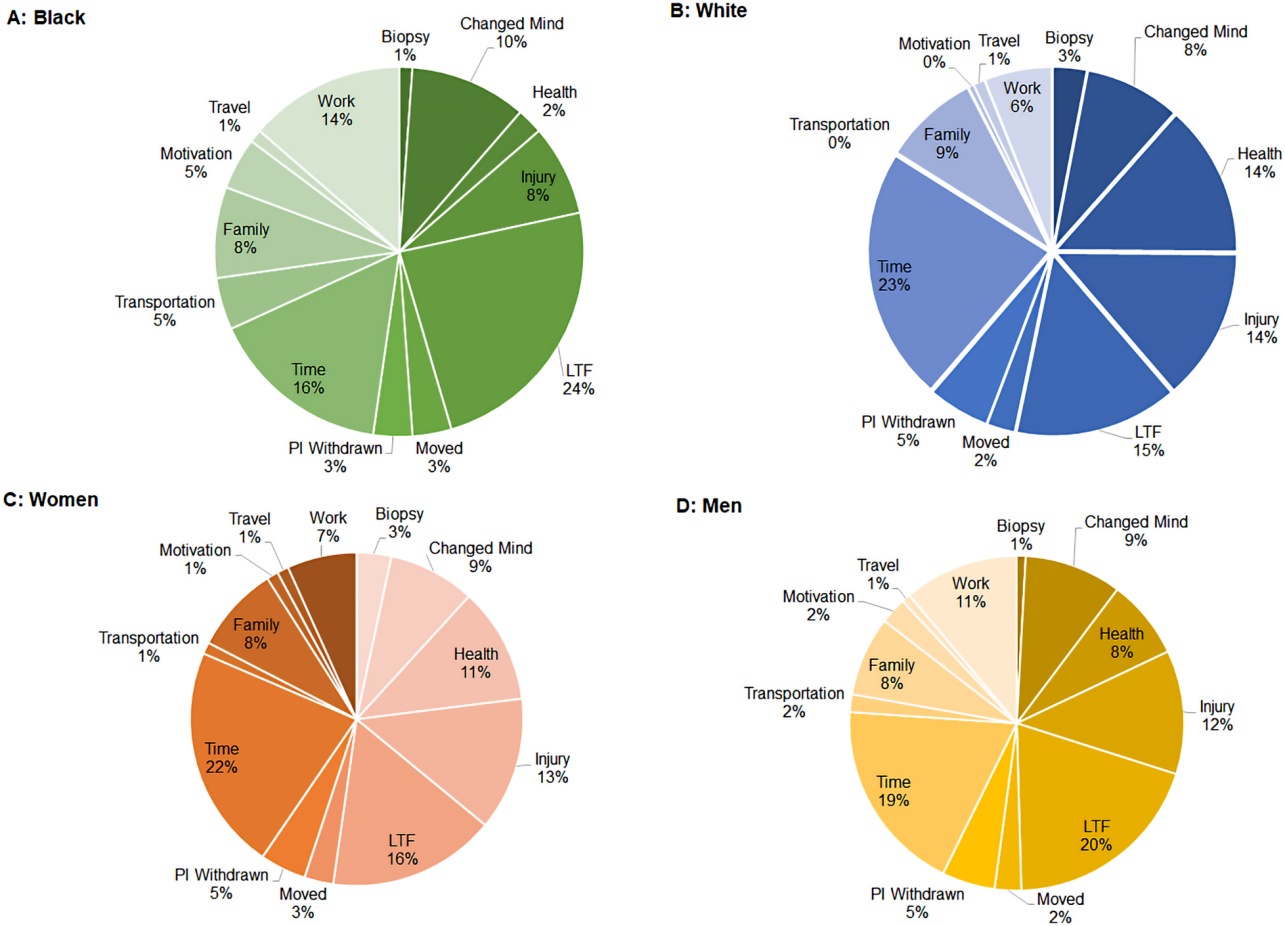
Proportion of participants by each dropout determinant. (Panel **A**) Black participants; (Panel **B**) White participants; (Panel **C**) Women participants; (Panel **D**) Men participants.

Figure 1 Panels A and B display the proportion of Black and White participants who fall into each dropout determinant category. Black participants were more likely to dropout because of work responsibilities (Black: 13.6% vs. White: 6.0%), were lost to follow-up (23.9% vs. 14.6%), had transportation issues (4.6% vs. 0.0%), changed their minds (10.2% vs. 8.5%), or reported lack of motivation (4.5% vs. 0.5%). White participants were more likely to dropout because of lack of time (White: 22.6% vs. Black: 15.9%), reoccurring injury (13.6% vs. 8.0%), or new or reoccurring health issues (13.6% vs. 2.3%).

**Figure 2:**
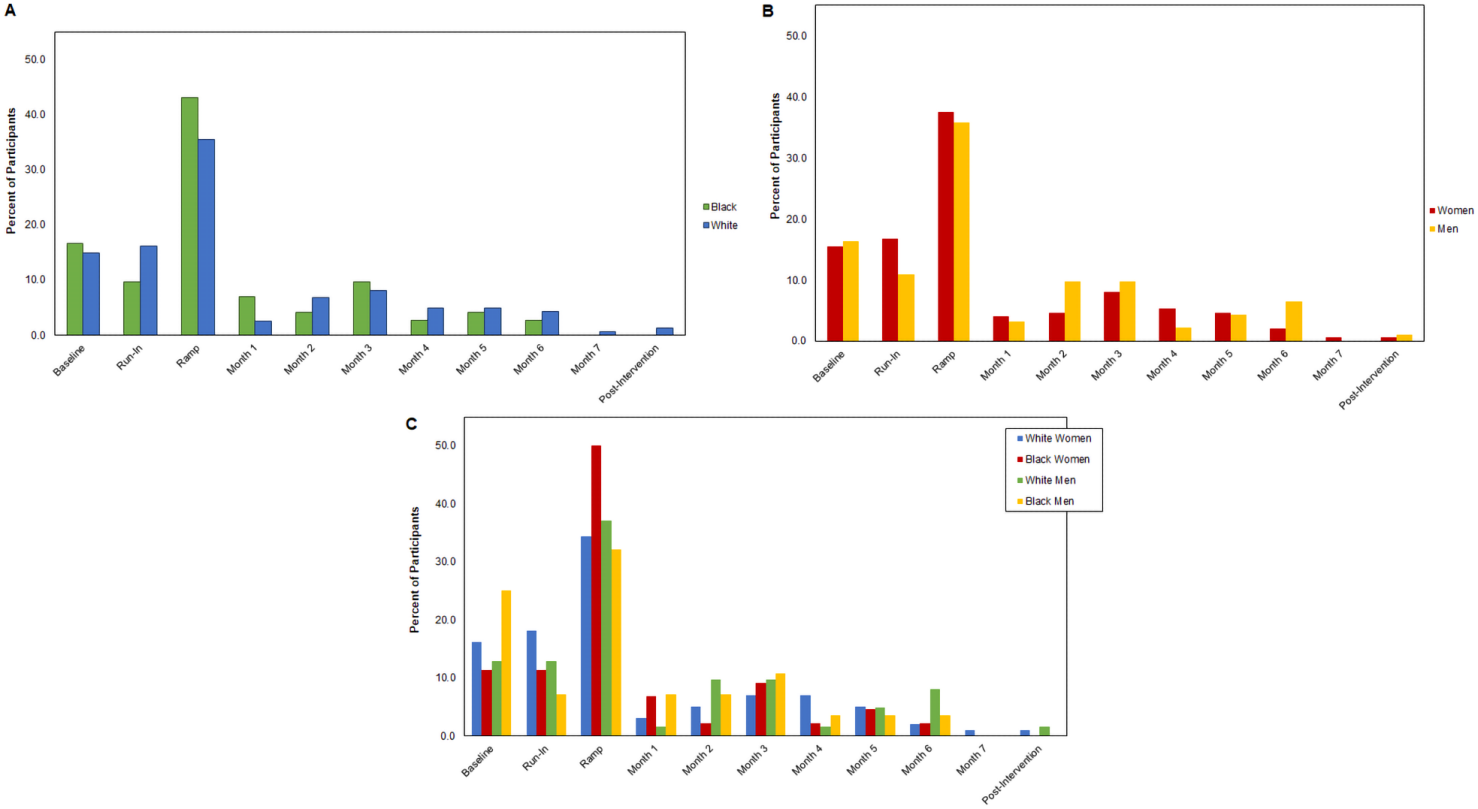
Proportion of participants by dropout timing category. (Panel **A**) Black and White participants; (Panel **B**) Men and women participants; (Panel **C**) White women, Black women, White men, and Black men participants.

Figure 2 Panel A displays the proportion of Black and White participants who fall into each dropout timing category. Compared to their Black counterparts, White participants were more likely to dropout during the run-in control period (White: 16.1% vs. Black: 9.7%) of the intervention. Compared to their White counterparts, Black participants were particularly more likely to dropout during the exercise ramp period (Black: 43.1% vs. White: 35.4%) of the intervention.

### Sex differences in dropout determinants and timing

Figure 1 Panels C and D display the proportion of men and women who fall into each dropout determinant category. Women were more likely to dropout due to lack of time (women: 21.9% vs. men: 18.8%), new or reoccurring health issues (11.2% vs. 7.7%), or family/caregiving responsibilities (8.4% vs. 7.7%). Dropout for men was more likely due to loss to follow-up (men: 19.7% vs. women: 16.3%), work responsibilities (11.1% vs. 6.7%), changed their minds (9.4% vs. 8.4%), or reported lack of motivation (2.7% vs. 1.1%).

Figure 2 Panel B displays the proportion of men and women who fall into each dropout timing category. Women were more likely to dropout during the run-in control period (women: 16.8% vs. men: 10.9%) or exercise ramp period (37.6% vs. 35.9%). Whereas, men were more likely to dropout during baseline assessment visits (men: 16.3% vs. women: 15.4%).

### Sex and race differences in dropout determinants and timing

**Figure 3:**
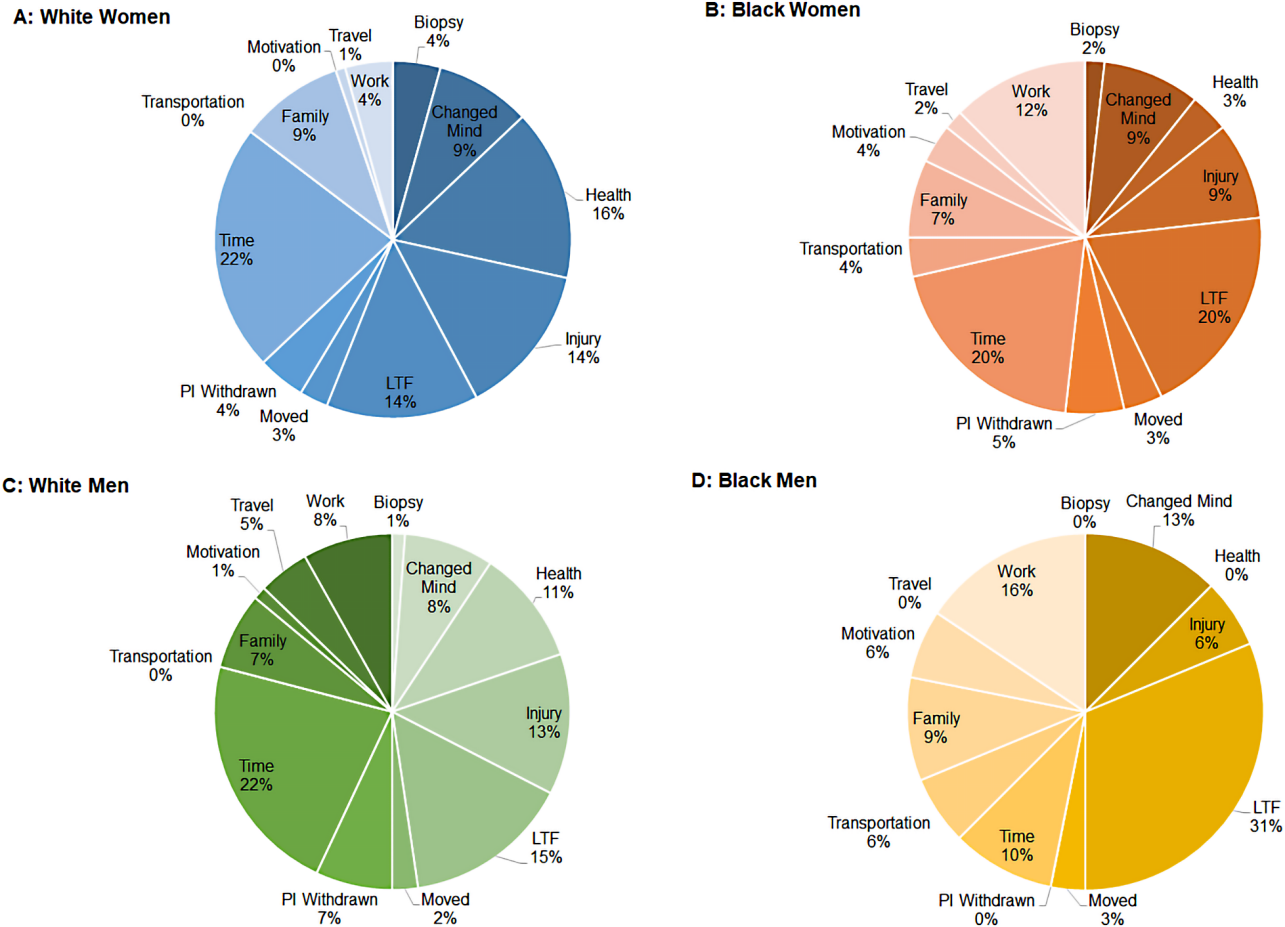
Proportion of participants who dropout across time by race and sex. (Panel **A**) White women; (Panel **B**) Black women; (Panel **C**) White men, (Panel **D**) Black men.

Figure 3 Panels A–D display the proportion of participants in each race and sex category who fall into each dropout determinant category. Dropout for Black men and women was more likely due to loss to follow-up (Black men: 31.3% and Black women: 19.6%), work responsibilities (15.6% and 12.5%), changed of mind (12.5% and 8.9%), transportation issues (6.3% and 3.6%), or reported lack of motivation (6.3% and 3.6%). Women in general reported lack of time more often than men as a reason for dropout (White women: 22.4% and Black women: 22.1%). Compared to both White men and Black women, White women (9.5%) and Black men (9.4%) reported family/caregiving responsibilities more often as a reason for dropout.

Figure 2 Panel C displays the proportion of participants in each race and sex category who fall into each dropout timing category. As compared to other race and sex subgroups, Black men (25.0%) dropped out more often during baseline assessments. White women (18.2%) dropped out more often during the run-in control period. Regardless of race and sex, most participants dropped out during the ramp period of the exercise intervention; with Black women (50.0%) and White men (37.1%) having the highest dropout rate during this period of the intervention.

## Discussion

The purpose of this secondary descriptive analysis was to characterize race and sex differences in dropout determinants and timing among previously sedentary adults with overweight or obesity enrolled in the STRRIDE trials. At baseline, Black men and women had greater body mass indexes compared to their White-counterparts, and women had lower cardiorespiratory fitness levels compared to men. These baseline differences draw attention towards understanding why some individuals who were previously sedentary with overweight or obesity may struggle to adopt an exercise or caloric restriction intervention compared to individuals with lower body mass and greater fitness levels. Taken together with our finding that most participants dropped out during the ramp period of the intervention, these characteristics potentially suggest Black men and women may benefit from a more gradual training titration, increasing the likelihood of successful progression to their target exercise prescription.

Little research has investigated race and sex specific differences in determinants and timing of dropout from exercise interventions with and without caloric restriction. The Diabetes Prevention Program evaluated 1,076 participants who were randomized to the intensive lifestyle intervention arm for barriers (i.e., “determinants”) to weight loss and physical activity goals (14). The most frequently reported barriers for achieving weight loss and physical activity goals were lack of self-monitoring, missed social cues, poor time management, internal cues such as mood, and lack of motivation. Specific to weight loss, the majority of the barriers were significantly associated with younger age, greater obesity, and non-White race/ethnicity. On the other hand, physical activity barriers were significantly associated with sex—being a woman—and greater obesity (14). While our study evaluated dropout during the entire study period—baseline assessments, intervention, post-assessments—the vast majority of dropout occurred during the active intervention allowing for a possible comparison. We found the most common reasons for dropout were lack of time (i.e., time management) and being lost to follow-up, however we did not have measures of internal or social cues to compare to the Diabetes Prevention Program analyses.

A retrospective study from the South Tyneside Exercise Referral Scheme assessed predictors of physical activity dropout over a 12-week period in individuals at risk for or who have already developed health conditions associated with physical inactivity (15). Among 6,894 participants who attended the initial consultation session, 50% dropped out from the Exercise Referral Scheme, with a majority dropping out within the first 6 weeks (15). Women and those of older age were more likely to dropout. In addition, drinking alcohol, citing a lack of motivation, and childcare responsibilities *decreased* the likelihood of dropout—a somewhat unexpected and unusual finding. Contrarily in the current study, we found participants reported lack of motivation and caregiving responsibilities as reasons for why they dropped out from the STRRIDE trials, which differs from the Exercise Referral Schemes analysis. One the other hand, we similarly found individuals who dropped out did so early on in the study period, predominantly during the ramp period of the intervention (7).

More specific to race and sex differences in dropout determinants and timing, we found women—regardless of race—reported lack of time as the primary reason for dropout. Compared to their White-counterparts, Black men and women were lost to follow-up more often. In addition, Black participants reported work responsibilities, changing their minds, transportation issues, and reported lack of motivation more often than their White-counterparts. Interestingly, Black men and White women reported family/caregiving responsibilities more often compared to their sex-counterparts. Compared to other participants, Black men dropped out the earliest in the study period—during baseline assessments. We found a majority of individuals dropped out prior to completing the first month of the exercise intervention, predominantly during the ramp period of the intervention—with the greatest number of individuals who dropped out during this period being Black women and White men.

These findings call to attention the need for developing and implementing more targeted retention strategies that address race and sex differences to mitigate dropout from lifestyle interventions. Culturally competent strategies, including sex-specific and racially inclusive materials, community partnerships, framing interventions within the culture of the participants, remote or virtual options, incentives such as gas coupons, and providing caregiving opportunities may be important approaches for future researchers to consider (16–21). Given the lack of trust the Black community may have with academic research as a whole, interventionists should aim to develop a strong rapport with participants with the goal of establishing a trusting and caring relationship (16, 22). Although interventions that include behavioral counseling or health coaching strategies are effective methods aiding in behavior change, whether these methods lead to sustained behavior change in the long-term, specifically among Black participants, is poorly understood and warrants future investigation (16). When trying to prevent dropout early on in the intervention period, researchers may want to consider collecting information regarding participants current barriers, support systems, motivational influences, ambivalence, and decisional balance constructs prior to intervention initiation with the goal of mitigating negative influences early on in the intervention (23–28). Additionally, when designing an exercise intervention, allowing for a longer and less intensive progression/ramp-up period may be beneficial for participants who find current exercise prescriptions too lofty or unattainable. A prolonged ramp period may help ease the discomfort a participant who was previously sedentary may feel during or following an exercise bout, as well as allow the individual additional time to alter their schedule to better accommodate work and child care responsibilities in a more gradual approach.

A potential limiting factor of this analysis is the lack of information on socioeconomical factors, such as education status. In general, socioeconomic status appears to negatively influence one's ability to successfully participate in an exercise and/or caloric restriction intervention (8, 29). Additionally, individuals with low socioeconomic status and race/ethnic minorities tend to be underrepresented in exercise and caloric restriction interventions (30–33). Although this retrospective analysis could not control for these important factors, future research should consider the impact these factors have on dropout—and whether they confound or contribute to dropout—from exercise and caloric restriction interventions.

This analysis does not come without limitations. The STRRIDE trials reflect supervised lifestyle intervention conditions, thus may not be as generalizable or representative of real-world situations surrounding exercise and diet program dropout. Each STRRIDE trial varied in study design, intervention duration, and inclusion of run-in and/or ramp periods, which may have influenced the results. This retrospective analysis is limited in its ability to explore factors other than those reported by the participants, which were predominately behavioral or environmental. Future research investigating dropout determinants should not only explore behavioral and environmental factors, but also a range of psychosocial and biological factors—such as personality and molecular determinants—that may predispose an individual's decision making (34). Importantly, a more comprehensive approach may help to generate a complete understanding of the behavior change process within the context of exercise and caloric restriction interventions. Last, the STRRIDE trials were not originally designed to assess race and sex differences in dropout behavior, allowing us to only conduct descriptive comparisons in this secondary analysis. Study designs in the future should plan for these important differences within the context of exercise and caloric restriction interventions, and power the studies accordingly.

In this retrospective analysis, we found descriptive differences in determinants and timing of dropout by race and sex among previously sedentary adults with overweight or obesity enrolled in the STRRIDE trials. Compared to their White-counterparts, Black men and women were lost to follow-up more often. In addition, Black participants reported work responsibilities, change of mind, transportation issues, and lack of motivation more often. Compared to other participants, Black men dropped out the earliest—during baseline assessments. A majority of individuals who dropped out did so prior to completing the first month of the exercise intervention, predominantly during the ramp period of the intervention; primarily driven by Black women and White men. To help mitigate the growing race and sex differences in obesity and physical inactivity prevalence rates, these findings highlight the importance for future interventions to incorporate targeted retention strategies that address race and sex-specific differences affecting lifestyle intervention dropout.

## Data Availability

The raw data supporting the conclusions of this article will be made available by the authors, without undue reservation, upon reasonable request.
